# Vetinformatics from functional genomics to drug discovery: Insights into decoding complex molecular mechanisms of livestock systems in veterinary science

**DOI:** 10.3389/fvets.2022.1008728

**Published:** 2022-11-11

**Authors:** Rajesh Kumar Pathak, Jun-Mo Kim

**Affiliations:** Department of Animal Science and Technology, Chung-Ang University, Anseong-si, South Korea

**Keywords:** vetinformatics, livestock systems, functional genomics, drug discovery, veterinary science

## Abstract

Having played important roles in human growth and development, livestock animals are regarded as integral parts of society. However, industrialization has depleted natural resources and exacerbated climate change worldwide, spurring the emergence of various diseases that reduce livestock productivity. Meanwhile, a growing human population demands sufficient food to meet their needs, necessitating innovations in veterinary sciences that increase productivity both quantitatively and qualitatively. We have been able to address various challenges facing veterinary and farm systems with new scientific and technological advances, which might open new opportunities for research. Recent breakthroughs in multi-omics platforms have produced a wealth of genetic and genomic data for livestock that must be converted into knowledge for breeding, disease prevention and management, productivity, and sustainability. Vetinformatics is regarded as a new bioinformatics research concept or approach that is revolutionizing the field of veterinary science. It employs an interdisciplinary approach to understand the complex molecular mechanisms of animal systems in order to expedite veterinary research, ensuring food and nutritional security. This review article highlights the background, recent advances, challenges, opportunities, and application of vetinformatics for quality veterinary services.

## Introduction

Livestock animals are an essential part of our life. Science-led innovation in veterinary research that benefits both people and animals as individuals and populations is crucial to maintaining public health (1, 2). This encompasses research on fundamental animal biology and animal welfare, as well as disease prevention, diagnosis, and therapy. Such innovation offers several opportunities for improving animal and human health (3, 4). Currently, veterinarians face many challenges exacerbated by climate change, including the emergence of new diseases, as well as those of a rapidly growing human population that requires adequate food and nutrition. Therefore, integration of interdisciplinary approaches with veterinary science is urgently needed to decode the complex molecular mechanisms of livestock systems (5–7).

The functioning of livestock systems is an area of active, ongoing research. Advancements in mathematical science, statistical methods, computer science, and information technology help biologists learn about biological systems quantitatively and qualitatively (8). Computers are essential components of these scientific advancements, as they play a crucial role in research and development sectors and become a major tool for researchers. In the era of “omics,” we can easily handle big data using computers, but the term “bioinformatics” was not introduced until the beginning of the 1970s by Hogeweg and Ben Hesper, when DNA could not yet be sequenced (9, 10). DNA's role as genetic material was also a matter of debate before 1952. Avery et al. (11) demonstrated that a non-virulent bacterial strain could acquire virulence by absorbing purified DNA from a virulent strain (8). However, the scientific community did not immediately accept their findings. Many scientists instead believed that proteins, rather than DNA, were carriers of genetic information (8, 12). Hershey and Chase established the role of DNA as a genetic information–encoding molecule in 1952 when they demonstrated that bacteriophage-infected bacterial cells ingest and transfer DNA rather than protein (13). At this time, DNA's primary role was understood, but little was known about how the DNA molecule was arranged. It was only known that its monomers (i.e., nucleotides) were present proportionately (14). The DNA double-helix structure was finally discovered by Watson and Crick (15). Despite this achievement, it would still be another 13 years before the genetic code was cracked, and another 25 years before the first DNA sequencing techniques were made accessible (16–18). Accordingly, DNA analysis using computational tools lagged ~2 decades behind the study of proteins, whose chemical makeup was already better understood than that of DNA (8).

Due to significant improvements in the crystallographic determination of protein structures (19), protein analysis was bioinformatics' starting point in Gauthier et al. (8). Insulin's sequence, or the arrangement of its amino acids, was the first protein sequence to be published (20). Additionally, numerous improvements in determining the structure and sequence of proteins were also reported (10, 21). The first bioinformatician was an American physical chemist named Margaret Dayhoff (1925–1983) who made significant contributions and used computational approaches in the study of biochemistry and protein sciences. She is referred to as the mother and father of bioinformatics (19, 20, 22).

Needleman and Wunsch created the first dynamic programming method for pairwise protein sequence alignment in 1978 (23). Since the early 1980s, multiple sequence alignment (MSA) algorithms have been emerging, facilitated by CLUSTAL software, which was introduced to MSA in 1988 (24, 25). Further, the concept of a mathematical framework for amino acid substitution was introduced by Dayhoff with the development of a point accepted mutation matrix (26). In the 1970s, DNA became more actively researched than proteins. Additionally, parallel developments in biology and computer science took place in the 1980 and 1990. Since the establishment of the National Center for Biotechnology Information (NCBI) in 1988 and the start of the Human Genome Project in 1990, bioinformatics has received significant attention and become an integral part of the analysis of the human genome (27–29). Further, bioinformatics emerged as a separate interdisciplinary subject for research and development in different areas of science and technology (10). Its approaches are extensively utilized in biomedical and pharmaceutical research. In recent years, the veterinary science community has sought to use these approaches in their research. Therefore, the concept of vetinformatics has been introduced as a branch of bioinformatics that focuses on livestock animals for quality veterinary services (5, 30).

**Figure 1:**
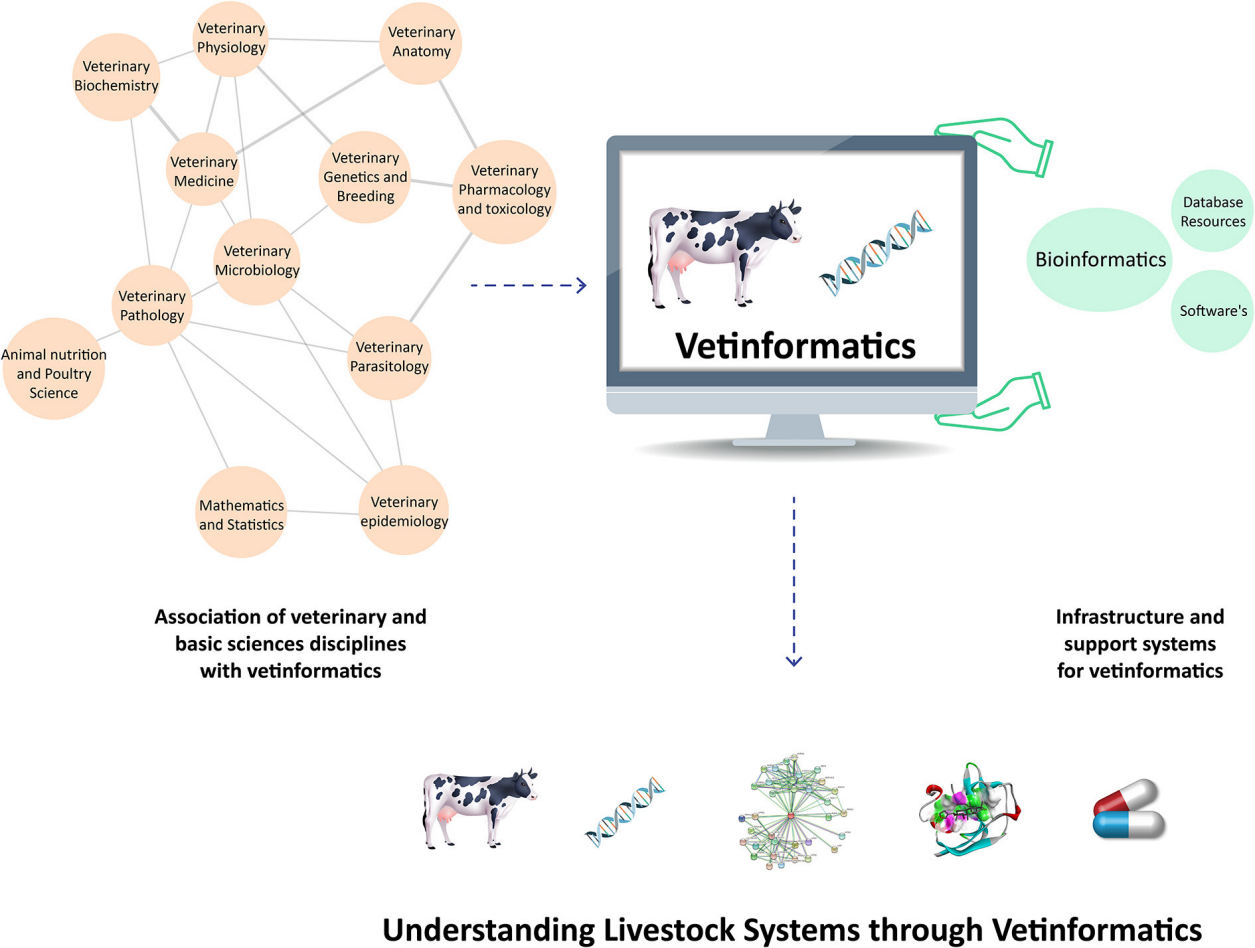
Integration of veterinary sciences, basic science disciplines, and support systems to create vetinformatics, enabling a better understanding of livestock systems in veterinary science.

In veterinary science, the livestock production system is a complex process that has three interconnected basic components: animal biology, the environment, and management techniques (31). Therefore, vetinformatics approaches are required to bridge the gaps between genotype and phenotype in order to improve livestock productivity and sustainability (5, 30). Large animal datasets have been produced as a result of improvements in various omics platforms and next-generation sequencing (NGS) technologies. Several bioinformatics databases and tools are available for their management and analysis, but these databases hold information about diverse groups of organisms (7, 10), and veterinarians require species-specific databases. Additionally, they require animal-based vetinformatics tools for data analysis and integration, as well as computational and mathematical models for analyzing animal behavior (32, 33). Accordingly, vetinformatics is required as a separate interdisciplinary subject to handle livestock data. By analyzing these large data sets, it is possible to accelerate research and development by extracting crucial information that enables researchers to understand livestock systems at molecular levels (Figure 1).

### Scientific disciplines linked with vetinformatics and their support systems

Vetinformatics is associated with the disciplines of veterinary sciences, basic sciences, and engineering; these disciplines provide infrastructure and an interdisciplinary nature to vetinformatics (5, 30, 33, 34). Several traditional and advanced subjects are associated with vetinformatics, such as veterinary physiology, biochemistry, anatomy, pharmacology and toxicology, parasitology, microbiology, pathology, epidemiology, genetics and breeding, and medicine, as well as animal nutrition and poultry science. Mathematical and statistical sciences also contribute to vetinformatics as basic-science disciplines. Computer science, information technology (IT), and computational resources serve as the foundation and support system for vetinformatics (5, 34). Accordingly, vetinformatics is created through the integration of veterinary sciences, basic sciences, and supporting disciplines. Vetinformatics uses computer science and IT to quickly provide solutions to complex challenges related to livestock systems (https://www.frontiersin.org/research-topics/33198/vetinformatics-an-insight-for-decoding-livestock-systems-through-in-silico-biology).

### Needs and aims of vetinformatics

In order to better understand livestock systems, vetinformatics is expanding and has contributed to the growth of research initiatives involving high-throughput DNA sequencing data analysis and other omics fields (https://www.veterinaryirelandjournal.com/ucd-research/165-how-omics-are-contributing-to-sustainable-animal-production; accessed on 14/7/2022). Vetinformatics' aim is to decode the enormous quantity of multi-omics data produced by high-throughput technologies, structural and functional characterizations of key genes and proteins, and visualizations of key components linked with livestock productivity and sustainability (7).

In general, the goals of vetinformatics include building databases that document information on medicinal plants, particularly for the development of herbal veterinary medicines *via* screening of phytochemicals against molecular drug targets using molecular docking (5, 6, 35). However, vetinformatics' aims also include collecting animal genetic resources in databases, developing these databases for managing omics data sets that are species- or organism-specific, and enhancing the content of existing veterinary databases so that they can be used more effectively (36–38). In addition, developing platform-independent graphical user interface–based software for integration and analysis of multi-omics data (10, 32–34), and improving the accessibility of public software for veterinary biotechnologists, scientists, and veterinarians would further vetinformatics' missions. Finally, vetinformatics also strives to educate undergraduate students, graduate students, and faculty of veterinary and animal sciences about the use of vetinformatics for the analysis of multi-omics data [(32, 34) https://www.frontiersin.org/research-topics/33198/vetinformatics-an-insight-for-decoding-livestock-systems-through-in-silico-biology].

## Recent advances in vetinformatics

The integration of omics science and technology with veterinary science opens exciting opportunities to decode livestock systems (7, 36, 39). Many animal genomes have been sequenced, and others are currently under sequencing and analysis. Although multi-omics data are generated regularly, more research is still needed to increase the efficiency and standardize interpretation, analysis, and integration of these data (7, 40). Additionally, the availability of big data in veterinary science has helped in the design of innovative algorithms and improved knowledge of cellular and phenotypic mechanisms [(7) https://ivcjournal.com/ai-the-newest-tool-in-veterinary-science/; accessed July 14, 2022]. Some animal-specific databases have already been developed to provide updated information to the veterinary science community (4, 37). Further, the use of machine and deep learning approaches in livestock research is reshaping the field in unanticipated ways. Exciting, cutting-edge models that connect genotype and phenotype allow the field of vetinformatics to grow quickly in the digital era and improve livestock productivity (41, 42).

## Challenges in vetinformatics

Vetinformatics is connected to veterinary sciences, basic sciences, and technology. Due to its interdisciplinary nature, vetinformatics may include people with a background in veterinary science or biology with little interest in computer programming, or people with a background in computer science who are unfamiliar with certain biological concepts. Due to the importance and application of vetinformatics in livestock research, many post-graduate programs in veterinary and animal science require exposure to the subject of vetinformatics. These programs may develop student interest in this emerging and interdisciplinary field, filling an urgent need for more researchers in vetinformatics. The major challenges faced by vetinformatics include managing big data in veterinary sciences; developing species-specific databases and tools for livestock research; improving the accuracy of available tools; developing novel algorithms and tools; analyzing and integrating multi-omics data; and identifying molecules for the development of drugs for treating livestock diseases (43, 44).

## Applications of vetinformatics in health, productivity, and sustainability of livestock

The scientific community produces complex data daily by using advanced molecular biology and biotechnology-based techniques (45, 46). These techniques require statistical approaches to quickly and accurately interpret these large-scale data (7, 40). Computational studies are the only method for analysis and interpretation of genome sequencing, assembly and alignment, differentially expressed genes, biological networks, protein modeling as well as molecular docking. The integration of such data is made possible by statistical and mathematical modeling approaches (5, 6, 10, 47, 48). Vetinformatics has tremendous potential to address challenging issues in veterinary science and related fields. Today, it is a vital tool for scientists and is crucial to the study of livestock. The following sections highlight the applications of vetinformatics.

### Assembly and annotation of newly sequenced genomes

Sequencing an animal's genome is necessary to understand the intricacy among them (49, 50). Aligning and combining fragments of genome sequences obtained from sequencing platforms is referred to as assembly. Depending on whether a reference genome is available or not, assembly can be divided into two categories: *de novo* assembly and reference-based assembly (50, 51). Genome assembly is essential for determining how gene structure and function will affect an organism's behavior. SPAdes is a highly cited genome assembler originally designed for small genomes. It was tested on microorganisms including bacteria, fungi, and other small genomes (52). Besides, it includes various assembly pipelines such as metaSPAdes, plasmidSPAdes, rnaSPAdes, truSPAdes, and dipSPAdes. These pipelines are useful for metagenomic data sets, assembly of plasmids from WGS data, *de novo* assembly of RNA-Seq data, barcode assembly, and highly polymorphic diploid genomes (https://cab.spbu.ru/files/release3.12.0/manual.html). In the field of genomics, annotating genomes through MAKER is convenient and easy. Genomes of eukaryotes and prokaryotes can be annotated independently and genome databases can be created using it. It is designed to identify repeats, align ESTs and proteins with the genomes, and produce *ab-initio* gene predictions (53).

Using high-throughput sequencing platforms, we now have sequenced genomes for many major animal species (54, 55). Zimin et al. (56) used a combination of whole-genome shotgun sequencing and hierarchical sequencing techniques to sequence the genome of the domestic cow (*Bos taurus*). They assembled the 35 million sequence reads to produce an improved assembly of 2.86 billion base pairs. Numerous computational tools can be used to further evaluate sequenced genomes. Several pipelines, resources, and software are available for computational assembly and study of the genome. Recent functional annotation of three domestic animal genomes (cattle, chicken, and pig) provides a useful resource for livestock research (57). The study of the genome is pertinent to many areas of research, including ancestry determination, genomic selection, and vaccine and drug design (58–60).

#### Transcriptome and RNA-Seq data analysis for studying gene expression

RNA-Seq has emerged as an effective approach for transcriptome analyses that will eventually make microarrays outdated for analysis of gene expression data (61). The field of research on gene expression has undergone a recent revolution. This technology has made possible the measure of simultaneous gene expression, enabling the discovery of candidate genes with potential biological significance (62). RNA-Seq analysis of porcine ovaries revealed 4,414 deferentially expressed genes and helped to discover their roles in the late metestrus and diestrus phases of the estrous cycle (48). The findings from a separate transcriptome analysis strongly suggested that IGF1, PGR, ITPR1, and CHRM3 regulate oocyte maturation and smooth muscle contraction in pigs, and provided direction for future research involving effective animal breeding programs (63).

Non-coding RNAs such as small interfering RNAs (siRNAs) and microRNAs (miRNA) play vital roles in gene regulation (64). Recent investigations have demonstrated that they are effective in treating a variety of diseases, and working as a biomarker for effective therapies (64–66). The role of miRNAs has been examined in several studies with respect to livestock diseases (66). Several candidate genes and miRNAs have been identified that could be helpful in treating mastitis disease in cows (67, 68). Vetinformatics-based approaches are useful in detection of siRNAs in host and their targets in pathogen genomes, leading to the development of novel treatments against livestock diseases (64, 69).

#### Metagenomic analysis for dissecting microbial communities and their role in livestock

Metagenomics allows for direct access to genetic information of whole communities by utilizing a variety of genomic technologies and computational approaches (39). It presents a considerably more comprehensive description than phylogenetic surveys because it enables access to the functional gene composition of microbial communities. Metagenomics provides information on potentially novel enzymes or biocatalysts, relationships between phylogeny and function for uncultured organisms, and evolutionary profiles of community structure and function (39). Kumar et al. (70) analyzed metagenomic data of bacterial communities in pig slurries, enhancing knowledge of how microbial abundance in swine slurries varies over time. Another microbiome analysis characterized changes in microbial community composition that resulted from feeding dairy cows one of two common diets: pasture and total mixed ration. Studies such as this one will contribute to the management of cattle feed and the study of rumen microbial ecology (71). On larger scales, metagenomics-based analyses will help to improve animal health, leading to enhanced livestock productivity and sustainability.

#### Sequence alignment and analysis for identification of biologically significant regions

With the availability of the BLAST tool beginning in 1990, sequence analysis has emerged as a key area of research (72). The field of sequence analysis is fairly broad, but in this section, we will focus on the analysis of nucleotide or protein sequences. A variety of sequence-alignment tools such as BLAST, FASTA, and Muscle are available to identify or compare two (pair-wise alignment) or more (multiple sequence alignment; MSA) sequences (10). Ajayi et al. (73) identified 67 genes in the bovine genome belonging to heat shock protein families using sequence alignment and analysis. Using *in silico* analysis, the study investigated transcription start sites and promoter regions of olfactory receptors in cattle, identifying five candidate motifs (MOR1, MOR2, MOR3, MOR4, and MOR5) important in gene regulation (74). It is also used to annotate recently discovered sequences, identify conserved and regulatory regions, and predict sequence physicochemical properties (10).

#### Molecular phylogeny for analyzing relationship among organisms

Another crucial area of research in vetinformatics is molecular phylogenetic analysis (75). Widely employed in evolutionary biology, molecular phylogenetic analysis can identify similarities between various animal sequences in order to infer their evolutionary relationship (76). Additionally, it facilitates the identification of critical elements in individual sequences and their association with other sequences, thereby playing an important role in drug and vaccine design (10, 76). In the field of molecular phylogeny, the Molecular Evolutionary Genetic Analysis (MEGA) (77) and PHYLIP (78) are well-known software. Besides, several other web-based tools are also available such as Clustal Omega (https://www.ebi.ac.uk/Tools/msa/clustalo/), MUSCLE (https://www.ebi.ac.uk/Tools/msa/muscle/), and T-Coffee (https://www.ebi.ac.uk/Tools/msa/tcoffee/) to perform multiple sequence alignment and building a phylogenetic tree using different methods. There has been increasing interest in reconstructing phylogenetic trees in biological science, and questions are being raised regarding the degree of confidence one should place in any given phylogenetic tree. The concept of bootstrapping and jackknifing was introduced to construct error-free phylogenetic tree (79). Phylogenetic analysis software like MEGA facilitates researchers to set a bootstrap value during phylogenetic tree reconstruction to confirm their accuracy (https://www.megasoftware.net/web_help_11/Bootstrap_Test_of_Phylogeny.htm). The Interactive Tree Of Life, i.e., iTOL (https://itol.embl.de/) and Tree View are highly cited tools facilitating phylogenetic tree visualization (80, 81). The bovine hepacivirus (BovHepV) of five positive samples that formed a separate branch from other BovHepV in a phylogenetic analysis conducted by Deng et al. based on the partial NS3 coding sequence (82). The findings suggested that these new BovHepV represent novel and emerging strains. Another study that conducted a molecular characterization and phylogenetic analysis of the lumpy skin disease virus (LSDV) that is circulating in northern Thailand revealed a relationship with other LSDVs. The LSDV that was isolated from northern Thailand shared genetic traits with the LSDVs that are currently circulating in China, Hong Kong, and Vietnam. This finding will be instrumental in developing disease control strategies against LSDVs (83).

### Genome wide association study for identification of important genomic regions

Genome-wide association study, commonly known as GWAS, is a powerful approach used to identify genetic variants linked to increased likelihood of a certain disease or trait (84, 85). The approach requires examining a large number of individual genomes in search of genetic variants that are more prevalent in individuals with a particular disease or trait. Once such genetic variants have been discovered, they are often utilized to look for neighboring variants that directly contribute to the disease or trait (84, 85). An analysis of GWAS can be conducted using single-locus, and multi-locus models (86). The General linear model (GLM), Mixed linear model (MLM), Logistic mixed model (LMM), and Compressed mixed linear model (CMLM) are the single locus models (87–89), and multi-locus model includes Multilocus random SNP effect mixed linear models (mrMLM) and Fast multilocus random SNP effect efficient mixed-model association (FASTmrEMMA) (86, 90–92). The computer programs commonly used for GWAS include PLINK (93), GenABEL (94), GenAMap (95), and GEMMA (96). In addition, the genomic databases and genome browsers such as NCBI (https://www.ncbi.nlm.nih.gov/), Animal QTLdb (https://www.animalgenome.org/cgi-bin/QTLdb/index), NAGRP (https://www.animalgenome.org/), Ensembl (https://asia.ensembl.org/index.html), and UCSC (https://genome.ucsc.edu/) are valuable resources (86). Besides, the Genome Analysis Toolkit (GATK) pipeline is an important platform for high-throughput genomics data analysis (97). There are a variety of tools available through GATK, most of which are focused on discovering variants and used for genotyping (https://gatk.broadinstitute.org/hc/en-us). Uemoto et al. identified six significant quantitative trait loci for immune-related traits in pigs affected by mycoplasma pneumonia of swine using GWAS, revealing novel insights into the genomic elements influencing pig production, respiratory illness, and immune-related traits (98). Another GWAS-based study identified candidate genes for milk production traits in Korean Holstein cattle and individual birth weight traits in Korean Yorkshire pigs (99, 100). Therefore, GWAS-based approaches have potential to decode important and complex traits linked with livestock productivity.

### Systems biology and integration of multi-omics data

Systems biology is a key subfield of vetinformatics and has made great contributions to the modeling and simulation of biological systems (101–103). The field aids in the integration of multi-omics data, including genomics, proteomics, metabolomics, and transcriptomics, in order to construct models that comprehensively characterize the behavior of biological systems under various conditions (104). In the past, researchers were forced to focus on single genes or proteins, but as omics technology and systems biology have advanced, the paradigm has changed from a reductionist approach to a holistic approach (104). Through network modeling and analysis, systems biology enables prediction of the behavior of whole systems and identification of essential components involved in various biological processes, both of which ultimately contribute to advancements in animal welfare and livestock productivity (101–104).

### Network biology and analysis

In network analysis, networks represent relationships among the components of a given system (104, 105). In biological systems, these relationships have attracted significant attention in recent years, founding the new interdisciplinary area called “network biology” (105). In network biology, biological systems are illustrated in the form of nodes and edges (105). Different types of networks such as signal transduction networks, protein-protein interaction networks, gene regulatory networks, and metabolic networks contain complex information about their relevant systems (104, 105). Nodes can represent genes, proteins, or metabolites, while edges represent interactions or relationships, according to the type of network (104). Network biology approaches are highly useful in investigations of hub nodes and drug targets, as well as identification of key components involved in regulating biological systems (47, 104, 106, 107).

### Protein structure modeling, visualization, and validation

It was once challenging to predict a protein's 3D structure from its amino acid sequence. Now, these predictions are facilitated by improvements in protein structure prediction methods as well as the development of AlphaFold, a deep learning–based tool for protein structure modeling (108–110). When the target protein structure cannot be elucidated by experimental techniques, computational approaches become extremely important (110). These approaches can be used to predict protein structure, and the predicted structure can be utilized in drug screening. Additionally, computational approaches are used for predicting protein-protein interactions, structural comparison, and alignment (110, 111). In addition, several tools have been developed for visualization, refinement, and validation of the predicted 3D protein model. PyMOL is most often used tool for visualization, while Swiss PDB viewer, Rampage, PROCHECK, and Structural Analysis and Verification Server are extensively used for evaluation and model validation (109, 112, 113). With use of these tools, we can improve the quality of predicted models for further research (https://saves.mbi.ucla.edu/). Pan et al. predicted the cow milk 3D structures of αs1-CA and β-CA using I-TASSER to understand its dynamics (114). Additional research has modeled protein structure using computational approaches for livestock therapeutics development (115–117).

### Binding site prediction

In drug discovery and design, binding site prediction is a crucial and significant step. A protein's 3D structure must be understood to identify amino acid residues present in the binding site. In order to learn more about the binding site and other sites, such as allosteric sites, computational tools are available to measure the area and volume of cavities in proteins (110). In vetinformatics, precise knowledge of the binding site is required to elucidate receptor–ligand interactions. Some molecular modeling and docking software packages offer the capability to predict and define the binding site prior to the docking simulation. Additionally, some web-based tools like CASTp and COACH are used for binding site predictions (118, 119).

### Drug discovery and design for the management of livestock disease

**Figure 2:**
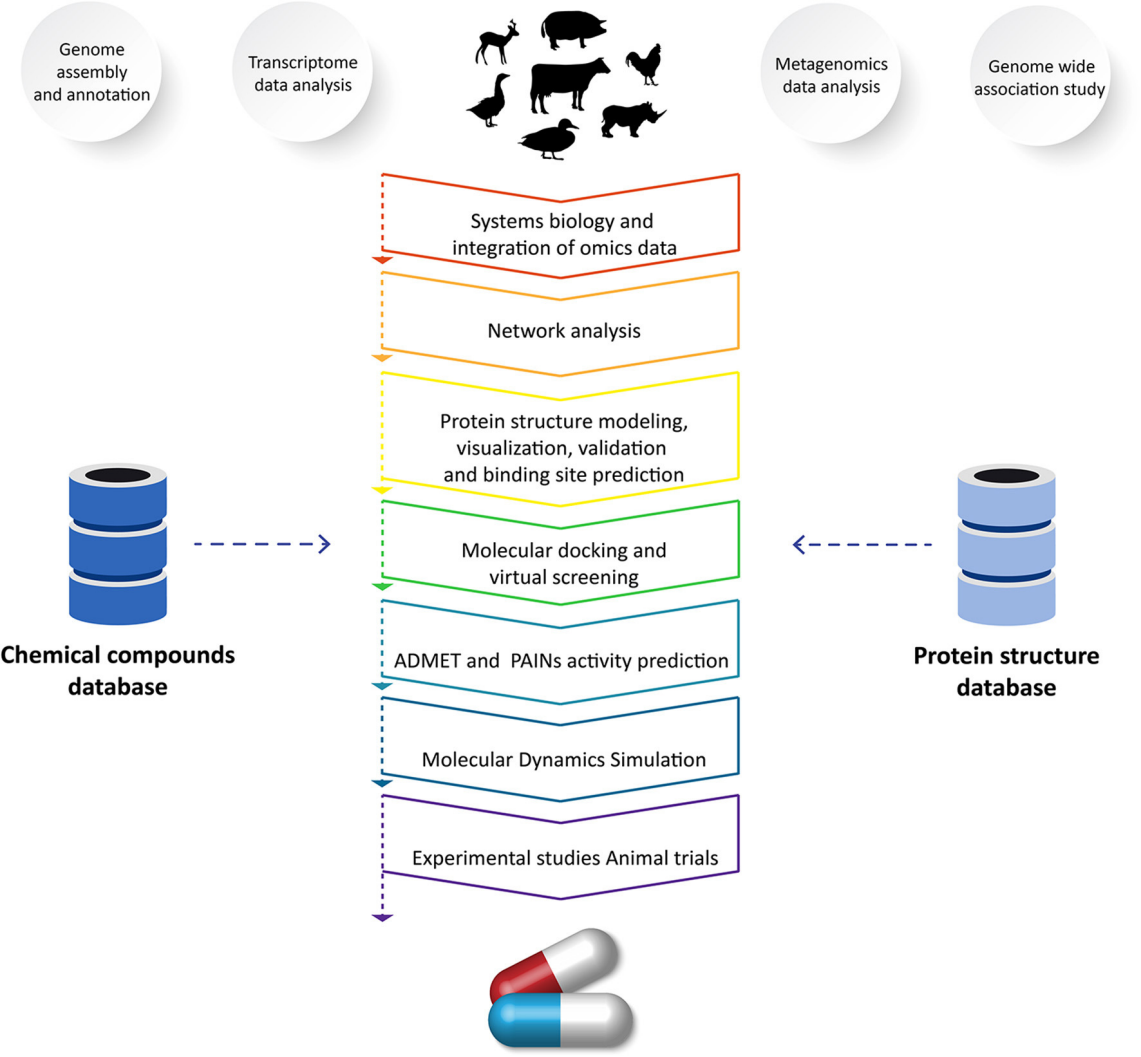
Application of vetinformatics to analyze high-throughput sequencing data for discovery of novel drug molecule(s) for veterinary application.

The emergence of novel diseases decreases livestock productivity and represents a pressing challenge in the field of veterinary science. Effective treatments are unavailable for many diseases (6). Therefore, there is an urgent need to use vetinformatics approaches to identify novel lead molecules for drug development (5). In the process of developing new drugs, computational methods act as a valuable resource (110, 120). Finding a small molecule that can geometrically and chemically fit in a cavity of a macromolecular target is the aim of computer-assisted drug discovery programs (109, 110). Recent developments in computational approaches have facilitated the estimation of receptor–ligand binding energy through molecular docking simulations, prediction of pharmacokinetics and pharmacodynamics, and optimization of lead molecules (121). Due to advancements in computer power and algorithms, the field of drug discovery and design has achieved significant progress. For developing models and tools for drug discovery and design, computational methods including the hidden markov model, artificial neural networks, support vector machines, and genetic algorithms are frequently employed (109, 110, 121). In order to accelerate the drug development process, several issues have been solved, leading to a major improvement in these approaches and tools to reduce the time and cost of drug discovery programs (5, 30, 109). Several approaches and methods that play significant roles in veterinary drug discovery programs are highlighted in the following sections (Figure 2).

#### Molecular docking and virtual screening for identification of lead compounds

Recent developments in computational approaches have made it possible to predict molecular receptor–ligand interactions in the bound or complex state with perfect accuracy (110, 122, 123). To predict the interaction of small compounds with macromolecular targets, software such as AutoDock, AutoDock Vina, Glide, and Discovery studio are available (109). These programs can be used to screen a wide range of prospective compounds, look for new compounds with specific binding properties, or test available medicines with functional group alterations using molecular docking and virtual screening (109, 110, 122, 124). Recently, *in silico* studies predicted the lead compounds for drug development against porcine reproductive and respiratory syndrome virus (PRRSV) *via* the screening of 97,999 natural compounds from the ZINC database (6). The compounds 7-deacetyl-7-oxogedunin, kulactone, and nimocin were also identified as potential multi-target leads for the inhibition of porcine CD163 scavenger receptor cysteine-rich domain 5 (CD163-SRCR5), as well as non-structural protein 4 (Nsp4) and Nsp10 of PRRSV (5). The inhibitors of the imidazole glycerophosphate dehydratase protein in *Staphylococcus xylosus* were also identified through virtual screening (117).

#### ADMET and PAINs activity prediction of lead compounds

The primary criteria for sorting ligands in drug discovery programs involve its absorption, distribution, metabolism, excretion, and toxicity prediction, or ADMET (109, 121). These criteria act as a fundamental standard for testing candidate molecules. It is widely believed that every drug discovery program should consider these criteria, or Lipinski's rule of five, to evaluate orally active drugs (123, 125). In the early stages of the drug discovery process, abiding by these criteria is crucial for finding the most appropriate drug-like compounds, and it considerably reduces the late-stage failure of candidate molecules during preclinical or clinical trials (109, 110). Additionally, we can filter molecules that are related to pan-assay interference compounds (PAINS). Instead of directly affecting a specific target, PAINS typically respond non-specifically with many biological targets. In order to prevent non-specific binding and toxicity, a filter should be used (126, 127). Therefore, it is recognized as a cost-effective and time-saving approach in veterinary drug discovery program (5, 109, 110).

#### Pharmacophore and quantitative structure–activity relationship modeling

A pharmacophore is a molecular framework containing essential features of a drug's active component. Pharmacophore modeling is extensively used in the development of novel compounds (109, 110, 128). It can be used to represent and distinguish molecules at a 2D or 3D level by schematically illustrating the essential components of molecular recognition (110, 123). Relatedly, quantitative structure–activity relationship modeling is a widely used drug discovery approach that utilizes a molecule's physicochemical properties to predict its biological activity (110, 129). Both of these approaches can be used to find novel treatments for livestock diseases (5, 30).

#### Molecular dynamics simulation of proteins and protein–ligand complexes to determine their dynamics and behavior during interactions

Molecular dynamics simulation is used to computationally visualize the movement and behavior of a molecular system at the atomic level (110, 130). It offers a wealth of knowledge regarding the interactions between proteins and ligands and provides complex structural information on macromolecular structures (109, 110). This knowledge is crucial for understanding the structure–function relationship among the target and its dynamics during protein–ligand interactions, ultimately supporting drug discovery processes (109). As a result, it is widely utilized to characterize protein–ligand interactions in modern drug discovery programs (6). Additionally, it is used to validate predicted protein models, understand the dynamics of protein folding and unfolding and protein–ligand dynamics, examine the effects of mutations on structures, and understand binding dynamics at other sites (5). A recent study described the role of DGAT1 missense non-synonymous single nucleotide polymorphisms (SNPs) in dairy cattle using computational approaches. The DGAT1 variants (W128R, W214R, C215G, P245R, and W459G) were analyzed initially through sequence- and structure-based tools, then evaluated using molecular dynamics simulation to understand their structural and conformational dynamics compared to wild-type structures and improve milk quality in cattle (47).

### Designing vaccines for livestock diseases

Emerging pathogens are a major threat to livestock productivity that requires the identification of vaccine candidates in order to ensure long-term protection of animals (33, 131). In order to provide broad-spectrum and long-term protection against different viral and bacterial diseases, new approaches to vaccine development must be created (10, 132). In the post-genome era, identifying specific antigenic regions to activate certain arms of the immune system was a major challenge (115, 133). To address this issue, computational vaccine design has been a major area of interest for researchers over the last two decades. Several tools and web-based resources have been developed that have proven useful in vaccine design (133, 134). Researchers can now utilize advanced vetinformatics approaches to design vaccines that provide protection against livestock diseases (33, 115, 131).

### Machine and deep learning approaches in livestock research

Machine and deep learning approaches have received significant attention from veterinary scientists (135, 136). Computers are equipped with an adaptive mechanism that enables them to learn from examples and experiences (137). Machine and deep learning provide information-processing capabilities for handling various types of real-life information (137). In order to make predictions or conclusions about target datasets, these algorithms often build mathematical models using sample datasets, also referred to as training datasets (137, 138). The recent advancements in artificial intelligence have made it even easier to analyze animal behavior in videos using machine vision and machine learning (139). The development of predictive models such as Convolutional Neural Network (CNN) and Long Short-Term Memory (LSTM) based on modern machine techniques are helpful in livestock research (139, 140). It was shown that the development of a recurrent neural network (RNN) model with an LSTM could classify cattle behavior in a reasonable manner (141). Recently, CNN and Bidirectional Long Short-Term Memory (BiLSTM) were used for video-based identification of individual cattle (140), and C3D-ConvLSTM (Convolutional 3D- Convolutional Long Short-Term Memory) based model was used for cow behavior classification over 86% accuracy (142).

Enabled by advances in omics, an enormous amount of biological data is produced every day, and these large data sets allow researchers to build machine learning models in order to make relevant predictions and minimize the expense and duration of experiments (137, 138, 143). These approaches play important roles in different areas of vetinformatics, such as gene discovery and genome annotation, gene expression analysis, drug target prediction, protein modeling, drug discovery, text mining, digital image processing, and helpful in precision livestock farming (137, 138, 143).

### Development of databases and tools for vetinformatics

Databases and tools related to veterinary science are essential for computer-based examinations of livestock data (30, 32, 33). Several databases and tools are available, but most databases contain information about many organisms (10) (Table 1). Due to recent developments in the area of vetinformatics, some animal-specific databases have been developed in recent years, but their availability is still insufficient (36–38). In the post-genomic era, large multi-omics data sets about livestock animals are urgently needed to develop species-specific databases to support veterinary science. Species-specific databases would help veterinary researchers easily find information about target animals. In addition, the availability of multi-omics data will help to improve the prediction, development, and accuracy of new algorithms that solve problems related to animal breeding, develop disease diagnostics, and offer solutions that increase livestock productivity and sustainability (138, 159–161). Some of the important software used for livestock research is highlighted in Table 2.

**Table 1:** List of important databases for research in vetinformatics.

**S. No**.	**Database**	**Application**	**Availability**	**References**
1	National Center for Biotechnology Information (NCBI)	Offers resources for research and development in different areas of the life sciences, including veterinary and animal sciences	https://www.ncbi.nlm.nih.gov/	(144)
2	Uniprot	Provides comprehensive resources related to protein sequences, including relevant functional and structural information	https://www.uniprot.org/	(145)
3	Pfam	Database of protein families used for domain analysis and related information	https://pfam.xfam.org/	(146)
4	Protein Data Bank (PDB)	Structural database with information on macromolecules' three-dimensional structures, which is useful in drug discovery and structural bioinformatics	https://www.rcsb.org/	(147)
5	AlphaFold Protein Structure Database	Database containing predicted 3D structures of human proteins and other key proteins	https://alphafold.ebi.ac.uk/	(148)
6	PubChem	NCBI Database of small molecules and related information, including the structure of chemical compounds, applicable for use in molecular docking and virtual screening	https://pubchem.ncbi.nlm.nih.gov/	(35)
7	ZINC	Database of commercially available molecules for use in virtual screening	https://zinc.docking.org/	(149)
8	GEO	Functional genomics database hosted at NCBI offering gene expression profiles that have been provided by an international scientific community	https://www.ncbi.nlm.nih.gov/geo/	(150)
9	Sequence Read Archive (SRA)	The largest collection of publicly accessible high-throughput sequencing data, comprising NGS data provided by the international scientific community for use in research and integration of multi-omics data	https://www.ncbi.nlm.nih.gov/sra	(151)
10	Kyoto Encyclopedia of Genes and Genomes (KEGG)	Database containing pathways for understanding biological systems	https://www.genome.jp/kegg/	(152)
11	Search Tool for the Retrieval of Interacting Genes/Proteins (STRING)	Database containing information about protein–protein interactions derived from experimental, computational, and text-mining techniques	https://string-db.org/	(153)
12	BioModels	Collection of mathematical models in standard file formats for further analysis and integration of biological systems	https://www.ebi.ac.uk/biomodels/	(154)
13	Bovine Genome Database	Database providing genomics resources and tools for bovine research	https://bovinegenome.elsiklab.missouri.edu/#:~:text=The%20Bovine%20Genome%20Database%20supportsthereford%20cow%2C%20L1%20Dominette%2001449	(37)
14	Porcine Translational Research Database	Database providing genomic and proteomic information involving pigs	https://www.ars.usda.gov/northeast-area/beltsville-md-bhnrc/beltsville-human-nutrition-research-center/diet-genomics-and-immunology-laboratory/docs/dgil-porcine-translational-research-database/	(36)
15	Animal-ImputeDB	Database and resource for the study of animal genotype imputation	http://gong_lab.hzau.edu.cn/Animal_ImputeDB/#!/	(38)
16	SNPRBb	Database containing trait-specific SNP resources for *Bubalus bubalis*, including information on important genomic variants	http://cabgrid.res.in:8080/snprbb/home.php	(155)
17	BuffSatDb	Water buffalo (*Bubalus bubalis*) genome-wide microsatellite database	http://webapp.cabgrid.res.in/buffsatdb/index.html	(156)
18	National Animal Genome Research Program	Comprehensive resource for research in livestock genomics	https://www.animalgenome.org/	(4)
19	Chickspress	Gene expression database for chicken tissues	https://geneatlas.arl.arizona.edu/	(157)
20	Ensembl genome browser	Genome browser containing genomic information of several livestock animals	https://www.ensembl.org/index.html	(158)

**Table 2:** A list of popular computational software available for livestock research.

**S. No**.	**Software**	**Application**	**Availability**	**References**
1	Basic Local Alignment Search Tool (BLAST)	Finds homologous and paralogous sequences and provides similarity searching	https://blast.ncbi.nlm.nih.gov/Blast.cgi	(72)
2	SRA Toolkit	Creating FASTQ files from SRA	https://github.com/ncbi/sra-tools/wiki/01.-Downloading-SRA-Toolkit	(162, 163)
3	FastQC	Assesses the quality of raw sequencing data produced by NGS platforms	https://www.bioinformatics.babraham.ac.uk/projects/fastqc/	(164)
4	Trimmomatic	Trims reads for Illumina NGS data	http://www.usadellab.org/cms/?page=trimmomatic	(165)
5	Cutadapt	Identifies primers, adapter sequences, poly-A tails, and other regions, and removes them from sequencing reads	https://cutadapt.readthedocs.io/en/stable/	(166)
6	fastp	Preprocessing of FASTQ files which includes quality control, adapter trimming, quality filtering etc	https://github.com/OpenGene/fastp	(167)
7	HISAT2	Maps next-generation sequencing reads quickly and accurately	http://daehwankimlab.github.io/hisat2/	(168)
8	Samtools	Used for post-processing of short DNA sequence read alignments	http://www.htslib.org/	(169)
9	Bowtie 2	Aligns sequencing reads to reference sequences	http://bowtie-bio.sourceforge.net/bowtie2/index.shtml	(170)
10	BWA	Mapping sequence reads to reference genome	https://bio-bwa.sourceforge.net/	(171)
11	Trinity	Assembles transcriptome or RNA-Seq data produced by the Illumina NGS platform using the *de novo* approach	https://github.com/trinityrnaseq/trinityrnaseq/wiki	(172)
12	edgeR	R package used to identify differentially expressed genes using RNA-Seq data	https://bioconductor.org/packages/release/bioc/html/edgeR.html	(173)
13	DESeq2	Differential gene expression analysis	https://bioconductor.org/packages/release/bioc/html/DESeq2.html	(174)
14	WGCNA	Co-expression network analysis	https://horvath.genetics.ucla.edu/html/CoexpressionNetwork/Rpackages/WGCNA/	(175)
15	GATK	Identification of variants using high-throughput sequencing data	yandell-lab.org/software/maker.html	(176)
16	Molecular Evolutionary Genetics Analysis (MEGA)	Creates phylogenetic trees and performs statistical analyses of molecular evolution	https://www.megasoftware.net/	(77)
17	Velvet	Handles *de novo* genome assembly using short-read sequencing data	https://www.ebi.ac.uk/~zerbino/velvet/	(177)
18	SPAdes	Single-cell and multi-cell genome assembly	https://cab.spbu.ru/software/spades/	(52)
19	MAKER	Genome annotation	https://github.com/Yandell-Lab/maker	(53)
20	REVIGO	Summarizes and visually represents gene ontology terms	http://revigo.irb.hr/	(178)
21	Multi-Experiment Viewer (WebMeV)	Creates analyses and visualizations of genomic data	https://webmev.tm4.org/#/about	(179)
22	Gene Set Enrichment Analysis (GSEA)	Facilitates the analysis and interpretation of gene expression data	https://www.gsea-msigdb.org/gsea/index.jsp	(180)
23	DIAMOND	Performs comparatively rapid sequence alignment of proteins or translated DNA sequences in order to examine of large amounts of sequence data	https://uni-tuebingen.de/fakultaeten/mathematisch-naturwissenschaftliche-fakultaet/fachbereiche/informatik/lehrstuehle/algorithms-in-bioinformatics/software/diamond/	(181)
24	Blast2GO	Used to perform genomic data annotation and gene ontology analysis	https://www.blast2go.com/	(182)
25	Cytoscape	Offers tools and plugins for visualization and research in network science and network biology	https://cytoscape.org/	(183)
26	AlphaFold 2	Uses deep learning methods to predict protein structure using amino acid sequences	https://github.com/deepmind/alphafold	(108)
27	PyMOL	Offers tools for the visualization and analysis of macromolecular structures in 3D	https://pymol.org/2/	(112)
28	Swiss PDB Viewer	Enables simultaneous analysis of protein structures, including calculation of H-bonds, angles, and atom distances as well as comparison and alignment of macromolecular structures	https://spdbv.vital-it.ch/	(113)
29	Chimera	Offers tools for visualizing and analyzing molecular structures and creating density maps, motions, and sequence and structural alignments, producing high-quality images	https://www.cgl.ucsf.edu/chimera/	(184)
30	Protein Variation Effect Analyzer (PROVEAN)	Predicts how an amino acid substitution or indel may affect the biological function of a protein	http://provean.jcvi.org/index.php	(185)
31	MarvinSketch	Offers tools for the conversion of structural file formats as well as for drawing, editing, importing, and exporting chemical structures and calculating their properties	https://chemaxon.com/products/marvin	(186)
32	CASTp	Binding site prediction	http://sts.bioe.uic.edu/castp/index.html?2was	(119)
33	AutoDock	Offers tools for molecular docking studies	http://autodock.scripps.edu/	(124)
34	SwissADME	Physicochemical properties, Pharmacokinetics, Druglikeness prediction	http://www.swissadme.ch/	(187)
35	GROningen MAchine for Chemical Simulations (GROMACS)	Offers high-performance molecular dynamics tools for simulations of proteins, lipids, and nucleic acids	http://www.gromacs.org/	(188)

## Future perspectives on vetinformatics

Since the beginning of the human genome project, the use of computers in biology has drawn significant interest and it is currently an essential tool in biological research. In the twenty-first century, it is difficult to imagine a novel discovery that does not rely on computational methods. Because computer software has been involved in most biological studies worldwide in the current omics era, many top research groups believe that integration of computers with biology has immense potential to decode complex biological systems, enabling the discovery of novel therapeutics and other useful information for the betterment of society. Therefore, vetinformatics will eventually become a crucial component of every veterinary science research lab. The management of big data in biology and veterinary science will also demand vetinformatics experts, who will reduce experimental work load and expense. As the human population grows, requiring commensurate increases in food production, it will be necessary to increase livestock productivity, advance animal breeding programs, improve the nutritional quality of animal products, and develop disease prevention and management strategies for animal welfare. This can be accomplished with the help of vetinformatics approaches that visualize the complexity of livestock systems in order to design solutions that meet our demands for higher livestock productivity.

## Conclusion

In recent years, vetinformatics has emerged as a vital subject and a popular interdisciplinary research area in veterinary sciences. The strength of vetinformatics and the ability of its methods to tackle challenging projects in veterinary sciences were highlighted in this review. Databases and other tools available for livestock research, along with their applications and availability, were also included. Vetinformatics approaches have proven their ability to resolve a variety of problems in veterinary science. To develop vetinformatics tools and databases that successfully target livestock systems for quality veterinary services, more resources need to be developed. Therefore, a conversation is needed in the veterinary science community that encourages the implementation of vetinformatics to understand livestock systems for the enhancement of animal welfare and drug discovery.

## Author contributions

J-MK and RKP developed the idea for this review article and its coverage. RKP wrote the manuscript. J-MK supervised the work and edited the manuscript. Both authors have read the final manuscript and approved the submission.

## Funding

This work was supported by the National Research Foundation of Korea (NRF) grant funded by the Korean government (MSIT) (NRF-2022R1A2C1005830).

## Conflict of interest

The authors declare that the research was conducted in the absence of any commercial or financial relationships that could be construed as a potential conflict of interest.

## Publisher's note

All claims expressed in this article are solely those of the authors and do not necessarily represent those of their affiliated organizations, or those of the publisher, the editors and the reviewers. Any product that may be evaluated in this article, or claim that may be made by its manufacturer, is not guaranteed or endorsed by the publisher.
